# Chloroplast DNA analysis of the invasive weed, Himalayan balsam (*Impatiens glandulifera*), in the British Isles

**DOI:** 10.1038/s41598-020-67871-0

**Published:** 2020-07-03

**Authors:** Daisuke Kurose, Kathryn M. Pollard, Carol A. Ellison

**Affiliations:** CABI-UK, Bakeham Lane, Egham, Surrey TW20 9TY UK

**Keywords:** Plant sciences, Plant molecular biology, Biogeography

## Abstract

*Impatiens glandulifera* or Himalayan balsam (HB), is an invasive alien weed throughout the British Isles (BI). Classical biological control of HB in the BI using a rust fungus from the Himalayan native range was implemented in 2014. However, not all HB populations are susceptible to the two rust strains currently released. Additional strains are needed that infect resistant populations in order to achieve successful control. These are best sourced from the historical collecting sites. A molecular analysis was conducted using six chloroplast DNA sequences from leaf material from across the BI and the native range. Herbarium samples collected in the Himalayas between 1881 and 1956 were also included. Phylogenetic analyses resulted in the separation of two distinct groups, one containing samples from the BI and the native range, and the other from the BI only; suggesting that HB was introduced into the BI on at least two occasions. The former group is composed of two subgroups, indicating a third introduction. Ten and 15 haplotypes were found in the introduced and native range respectively, and with two of these found in both regions. Results show where to focus future surveys in the native range to find more compatible rust strains.

## Introduction

*Impatiens glandulifera* Royle (Balsaminaceae), commonly known as Himalayan balsam (HB), is an annual plant native to the foothills of the Himalayas. Introduced as an ornamental due to its showy flowers, HB has become a troublesome invader across Europe and North America^1, 2^. The introduction of an invasive species often occurs over large temporal and spatial scales and, as a result introductions are often poorly documented and difficult to trace^3^. For HB, however, there is much consensus that it was first introduced into the Royal Botanic Gardens at Kew (UK) in 1839 from Kashmir^4^. Nevertheless, there is some dispute over the date of introduction with Tanner^5^ proposing that HB was introduced prior to 1839, since the collector, John Forbes Royle, then curator of the East India Company’s botanical garden in Saharanpur (Northern India), took up a post at King’s College London in 1837. However, Morgan^6^ states that the species was first introduced into England from Nepal. Regardless, since its introduction, HB has spread rapidly (both naturally and human-mediated) throughout the British Isles (BI), where it is now one of the most prevalent alien plant species. HB typically invades riparian habitats, but is also found in damp woodlands, on waste lands and along railway lines where it can have negative ecological and environmental impacts. Its prolific growth means that this species can rapidly form dense monospecific stands which can outcompete the native flora^7^ and decrease the diversity of above and below ground invertebrates^8^. Plants can lower the abundance of arbuscular mycorrhizal communities in the soil^9^ and, due to their high sugar nectar production, flowers can draw pollinators away from native species^10^. Being an annual species, regarded as the tallest in the UK, HB dies back in the autumn leaving dead plant material to be incorporated in the water body, as well as bare ground, increasing flood potential and bank erosion^11^.

In 2006, the UK Government initiated a classical biological control (CBC) programme against HB and a rust fungus, *Puccinia komarovii* var. *glanduliferae*, was prioritised due to its prevalence and high level of damage in the native range^12^. The culmination of eight years of research into the life-cycle and safety of the rust formed the basis of a Pest Risk Assessment, and a strain of the rust fungus from India was approved for release by the UK Government in 2014^13^. Field observations and inoculation studies revealed significant variation in the susceptibility of selected HB populations to the rust in England and Wales; many populations were either weakly susceptible or resistant to the Indian strain of the rust^13^. Fortuitously, a viable strain of the rust from Pakistan was retrieved from storage in liquid nitrogen and inoculation studies conducted under controlled conditions found that this strain was able to infect some of the populations resistant to the Indian strain. Following ministerial approval, the strain from Pakistan was released into the UK in 2017; the Pakistani strain performed well in the field at many of the previously resistant sites, with high levels of leaf infection recorded^14^. Whilst the release of the Pakistani rust strain significantly improved field infection across the UK, during pre-release screening, conducted under controlled glasshouse conditions, HB populations at 30–50% of sites selected for potential new releases between 2018 and 2019 were found to be either immune or resistant to infection by both rust strains^14^. It became necessary, therefore, to identify additional strains of the rust that can infect these resistant HB populations in order to better manage the weed invasions. Biotrophic plant pathogens, such as rusts, have co-evolved with their host plants, and often can only infect one species, and may even be specific to certain genotypes within that species^15^. An incompatible interaction between some weed populations in the invasive range and the introduced pathogen has hindered the success of a number of CBC programmes^16, 17^. In order to achieve the best host–pathogen match, and to successfully control HB in the BI, it is important that plant genotypes are matched to the most compatible rust strains. Logically, such strains should be sourced from areas where the original plant collections were made. It is crucial, therefore, to understand the genetic diversity of HB both in the BI and the native range in order to identify dominant genotypes present in the BI and to pinpoint those areas where these match with populations in the native range.

Molecular techniques offer a unique opportunity to enhance the success of CBC and, as such, they are now a prerequisite of many programmes^3, 18, 19^. The ability to gain insight into the introduction of a species—including information on where introductions originated from, how many times the species was introduced, and the distribution and occurrence of specific genotypes (genetic diversity)—is an invaluable tool which can reduce both the time and costs associated with identifying, matching and therefore prioritising natural enemies for such projects^20, 21^. These molecular techniques are now widely used as a tool in CBC programmes against invasive, non-native or alien weeds, where both herbivores and pathogens have been exploited as biological control agents^22–25^. Chloroplast DNA (cpDNA) has frequently been used as a phylogeographical marker for analysing weed invasions^22, 26, 27^. The typical maternal inheritance of cpDNA in angiosperms means that any genetic structure that was present in the original introductions may be retained, in comparison to nuclear markers that are subject to gene flow via both pollen and seed^22^.

There is much phenotypic variation observed between plants of HB, both within and among populations, which, according to Beerling and Perrins^4^, is determined by abiotic factors. Most obvious is the variation in flower colour, from pink to purple to white, which is observed throughout the BI. To date, a number of molecular studies of HB have been undertaken which all concluded that, despite being introduced into the UK multiple times, HB populations here and in mainland Europe have a relatively low level of genetic diversity, when compared to populations from the native range^28–30^. The level of genetic variation within and between populations of an invasive species has been shown to influence the potential success of biological control^21, 31–33^. In this instance, it is important, therefore, to not only include HB leaf samples from UK rust-release sites but also leaf samples from where the two rust strains were collected in the native range.

Our aims in this study are to: (1) clarify how many HB genotypes are present in the BI and understand the distribution of the most dominant genotypes in this region: (2) compare these results with plant samples from India and Pakistan in order to identify where these introductions originated: (3) identify genetically matched haplotypes, particularly for the most dominant genotypes, between the BI and the native range so that future surveys for additional strains of *P. komarovii* var. *glanduliferae* can be better targeted. The results of this study should improve the chances or a more rapid success of the CBC programme against this prolific and ecologically-damaging weed in the BI.

## Results

Our preliminary work showed that variability in the internal transcribed spacer regions, including the 5.8S rDNA (rDNA-ITS) was very low (data not shown), which is consistent with the previous research^30^. The six cpDNA regions (*trnL*-*trnF*, *atpB*-*rbcL*, *rps16* Intron, *trnG* Intron, *psbA*-*trnH*^(GUG)^ and *rpl32*-*trnL*^(UAG)^) were selected due to high variability. The aligned sequences of *trnL*-*trnF*, *atpB*-*rbcL*, *rps16* Intron, *trnG* Intron, *psbA*-*trnH*^(GUG)^ and *rpl32*-*trnL*^(UAG)^ were 906, 804, 813, 632, 397 and 1,047 bp in length, respectively. Out of the total of 4,599 bp, there were 42 variable sites including 30 substitutions and 12 indels (Table 1). Variable sites in each region were as follows: *trnL*-*trnF*, one substitution; *atpB*-*rbcL*, one substitution; *rps16* Intron, five substitutions and three indels; *trnG* Intron, three substitutions and three indels; *psbA*-*trnH*^(GUG)^, nine substitutions and two indels; *rpl32*-*trnL*^(UAG)^, 11 substitutions and four indels. Based on the variable sites as mentioned above, 23 haplotypes (A-W) (eight and 13 haplotypes unique to the introduced and native range, respectively, and two haplotypes which occur in both regions) were found across the 86 individuals (52 populations) of HB sampled. From the 24 populations where samples were analysed from two or more individuals, 22 were fixed for a single haplotype and only two populations in the introduced range were polymorphic with two haplotypes.

**Table 1:** Polymorphic sites in the aligned sequences of the six chloroplast DNA regions in the haplotypes from 52 populations of *Impatiens glandulifera* in the native and introduced range.

	*trnL-trnF*	*atpB-rbcL*	*rps16* Intron								*trnG* Intron						*psbA-trnH*											*rpl32-trnL*^*(UAG)*^														
Haplotype	260	74	104	123	195	425	532	533	699	703	9	256	266	278	280	431	22	134	141	146	175	179	180	181	182	186	253	6	128	169	220	288	353	354	366	380	381	382	383	384	522	530
A	A	A	G	G	T	–	–	T	T	C	A	–	–	–	T	A	T	–	–	A	C	T	G	G	T	A	T	A	G	T	T	C	–	–	T	T	A	T	C	C	T	C
B	A	A	G	G	T	–	–	T	T	C	A	–	–	–	T	A	T	–	–	A	C	T	G	G	T	A	T	A	G	T	T	C	A	–	T	T	A	T	C	C	T	C
C	A	A	G	G	T	–	–	T	T	C	A	–	–	–	T	A	T	A	–	A	C	T	G	G	T	A	T	A	G	T	T	C	–	–	T	T	A	T	C	C	T	C
D	A	A	G	G	T	–	–	T	T	C	T	–	T	–	T	C	T	–	–	A	C	T	G	G	T	A	T	G	G	A	–	C	A	–	–	T	A	T	C	C	T	C
E	A	C	G	G	T	–	–	T	T	C	T	–	T	–	T	A	T	A	–	A	C	T	G	G	T	A	T	A	G	A	T	C	A	–	–	T	A	T	C	C	T	C
F	A	C	G	G	T	–	–	–	T	C	T	–	T	–	T	A	T	A	–	A	C	T	G	G	T	A	T	A	G	A	T	C	A	–	–	T	A	T	C	C	T	C
G	A	C	G	G	T	–	–	T	T	C	T	–	T	–	T	A	T	A	–	A	C	T	G	G	T	A	T	A	G	A	T	C	A	–	–	T	A	T	C	C	T	G
H	A	C	G	G	T	–	–	T	T	C	T	–	T	–	T	A	T	A	–	C	C	T	G	G	T	A	T	A	G	A	T	C	A	–	–	T	A	T	C	C	T	C
I	A	C	G	G	T	–	–	T	T	C	T	–	T	–	T	A	T	A	–	A	C	T	G	G	T	A	T	A	T	A	T	C	–	–	T	T	A	T	C	C	T	C
J	A	C	G	G	T	–	–	T	T	C	T	–	–	–	T	A	G	A	–	A	C	T	G	G	T	A	T	A	G	A	T	C	–	–	–	T	A	T	C	C	T	C
K	A	C	G	G	C	–	–	T	A	T	T	–	–	–	T	A	G	A	–	A	C	T	G	G	T	A	T	A	G	A	T	C	–	–	–	T	A	T	C	C	T	C
M	A	C	G	G	T	–	–	T	T	C	T	–	–	–	T	A	T	A	–	A	C	T	G	G	T	A	T	A	G	A	T	C	A	–	–	T	A	T	C	C	T	C
N	A	C	G	G	T	5 bp in^a^	–	–	T	C	T	–	–	–	T	A	T	A	–	A	C	T	G	G	T	A	T	A	G	A	T	C	A	–	–	T	A	T	C	C	T	C
L	A	C	G	G	T	–	–	T	T	C	A	–	–	–	T	A	T	–	5 bp in^b^	A	C	T	G	G	T	A	T	A	G	T	T	C	–	–	T	T	A	T	C	C	T	C
O	A	C	G	G	T	–	–	T	T	C	A	–	–	–	T	A	T	A	–	A	C	T	G	G	T	A	T	A	G	A	T	C	A	–	–	T	A	T	C	C	T	C
P	A	C	G	G	T	–	–	T	T	C	A	–	–	–	T	A	T	–	–	A	C	T	G	G	T	A	T	A	G	T	T	C	–	–	T	T	A	T	C	C	T	C
Q	A	C	G	G	T	–	–	T	T	C	T	–	–	–	G	A	T	A	–	A	C	T	G	G	T	A	T	A	G	A	T	C	–	–	–	T	A	T	C	C	G	C
R	A	C	G	A	T	–	T	T	T	C	T	C	–	–	T	A	T	–	–	A	C	T	G	G	T	A	C	A	T	A	T	C	A	A	T	G	G	A	T	A	T	C
S	A	C	A	G	T	–	–	–	T	C	T	–	T	–	T	A	T	–	–	A	C	T	G	G	T	A	T	A	G	A	T	G	–	–	T	T	A	T	C	C	T	C
T	A	C	A	G	T	–	–	T	T	C	T	–	T	A	T	A	T	–	–	A	C	T	G	G	T	A	T	A	G	A	T	G	–	–	T	T	A	T	C	C	T	C
U	A	C	G	G	T	–	–	–	T	C	A	–	–	–	T	A	T	A	–	A	C	T	G	G	T	A	T	A	G	T	T	C	A	A	T	T	A	T	C	C	T	C
V	A	C	G	G	T	–	–	–	T	C	T	–	–	–	T	A	T	A	–	A	C	T	G	G	T	A	T	A	G	A	T	C	–	–	–	T	A	T	C	C	T	C
W	C	C	G	G	T	5 bp in^a^	–	–	T	C	T	–	T	–	T	A	T	A	–	A	T	A	C	C	A	G	T	A	G	A	T	C	–	–	–	T	A	T	C	C	T	C

^a^Indel; ACTAT.

^b^Indel; AGTAA.

**Figure 1:**
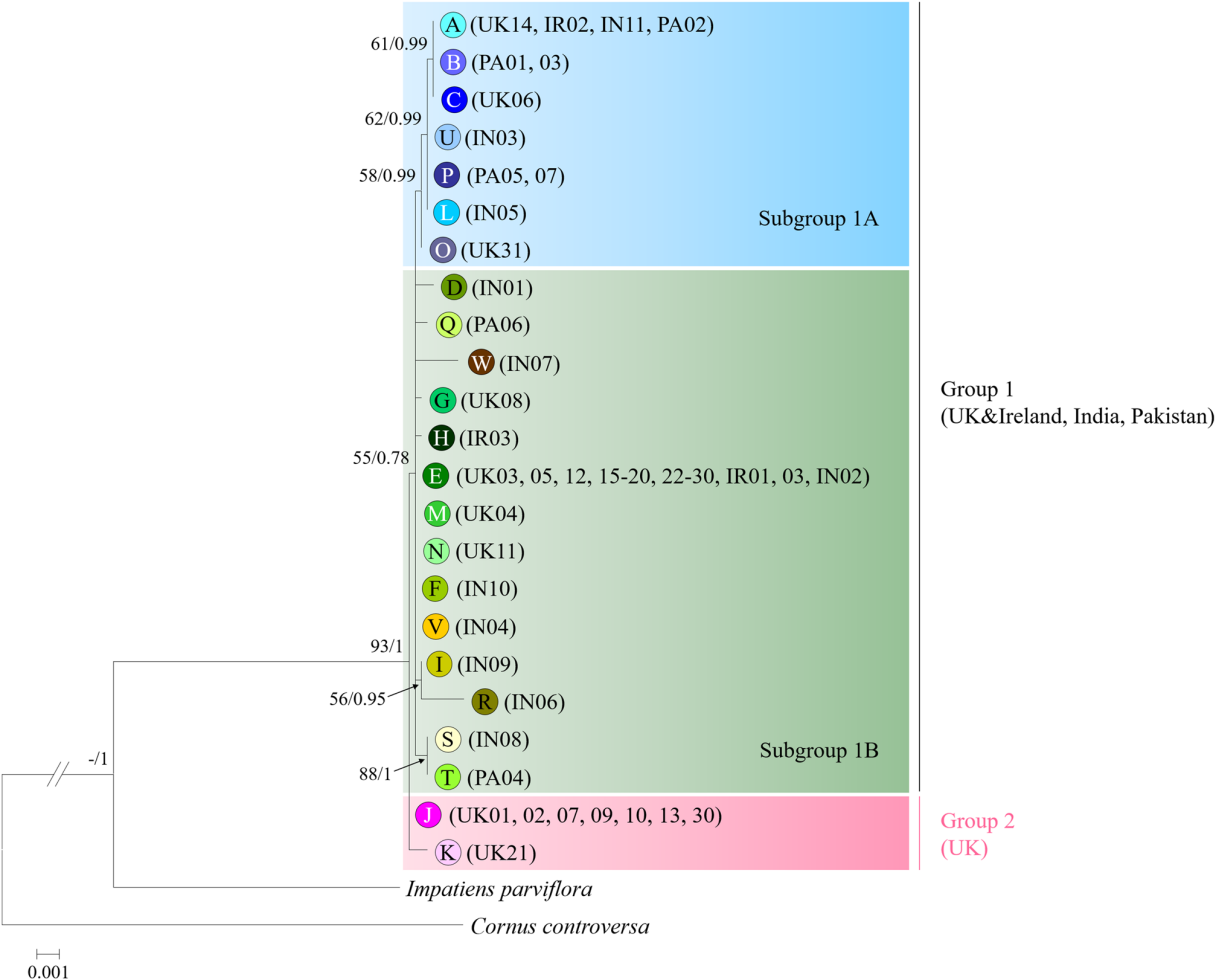
Phylogenetic tree based on sequence data of the six chloroplast DNA regions for *Impatiens glandulifera* haplotypes, as constructed by maximum-likelihood (ML) and Bayesian inference (BI) methods. The sequences of *Impatiens parviflora* and *Cornus controversa* was used as the outgroup species. Numbers above branches represent the bootstrap values (> 50% are shown) for ML (Left, 1,000 replicates) and BI analyses (Right).

A phylogenetic tree for HB was reconstructed by using the Maximum-likelihood (ML) and Bayesian inference (BIf) methods with the 23 different haplotype sequences of cpDNA, along with *Impatiens parviflora* and *Cornus controversa* as outgroups (Fig. 1). The ML tree was identical to that reconstructed by the BIf method. All the individuals of HB formed a monophyletic group with high bootstrap values and posterior probabilities. Based on the phylogenetic analyses, the 23 haplotypes could be divided into two groups, containing haplotypes from the introduced and native range (Group 1) and from the introduced range of the BI only (Group 2). Although Group 1 forms a monophyletic group, it could be divided into two subgroups (Subgroups 1A and 1B). The majority of samples from Pakistan are included in Subgroup 1A and those from India in Subgroup 1B (Table 2, Fig. 1). Haplotypes J and K belonging to Group 2 diverged earlier than Group 1 with a bootstrap value of 55%. There are examples from the seven samples from the Natural History Museum, collected from across England more than 100 years ago, in both of the groups and each of the subgroups.

**Table 2:** Site details of the Himalayan balsam populations from the British Isles, India and Pakistan used in the study.

Population no.	Location	Country	Latitude	Longitude	Altitude (m)	Collection year	*N*	Haplotypes	Group/subgroup^e^
**Introduced range**									
UK01	Killearn, Stirling	Scotland, UK	56.00297	− 4.35907	–	2016	2	J	2
UK02	Forth, South Lanarkshire	Scotland, UK	56.13263	− 3.93942	–	2016	2	J	2
UK03	River Till, Northumberland^a^	England, UK	55.67944	− 2.18283	–	2016	1	E	1B
UK04	Norham, Northumberland^a^	England, UK	55.73161	− 2.14806	–	2016	2	M	1B
UK05	River Tweed, Northumberland^a^	England, UK	55.67851	− 2.21306	–	2016	1	E	1B
UK06	Harrogate, North Yorkshire^b^	England, UK	54.36659	− 1.92535	–	1914	1	C	1A
UK07	Newbus Grange, Durham^a^	England, UK	54.48018	− 1.50706	–	2016	2	J	2
UK08	Colden Clough, West Yorkshire^a^	England, UK	53.74747	− 2.03029	–	2016	2	G	1B
UK09	Gorpley Clough, West Yorkshire^a^	England, UK	53.70696	− 2.12721	–	2016	2	J	2
UK10	Stanley Marsh, West Yorkshire^a^	England, UK	53.70769	− 1.47883	–	2016	2	J	2
UK11	Meanwood Grove, West Yorkshire^b^	England, UK	53.61275	− 1.51271	–	1908	1	N	1B
UK12	Boston, Lincolnshire^b^	England, UK	53.36102	− 0.23627	–	1945	1	E	1B
UK13	Framingham Pigot, Norfolk	England, UK	52.585	1.36972	–	2016	2	J	2
UK14	Harmondsworth Moor, Middlesex^a^	England, UK	51.493	− 0.48321	–	2016	8	A	1A
UK15	Silwood Park, Berkshire^a^	England, UK	51.40756	− 0.64374	–	2016	4	E	1B
UK16	Sunningdale, Berkshire^a^	England, UK	51.39426	− 0.63567	–	2016	2	E	1B
UK17	Bexley, Kent^a^	England, UK	51.44735	0.16217	–	2016	2	E	1B
UK18	Gatwick, West Sussex^a^	England, UK	51.15257	− 0.19838	–	2016	2	E	1B
UK19	Andover, Hampshire^b^	England, UK	51.20461	− 1.17105	–	1898	1	E	1B
UK20	Loughwood, Devon^b^	England, UK	50.92382	− 4.01043	–	1916	1	E	1B
UK21	Bovey Tracey, Devon^b^	England, UK	50.60359	− 3.64852	–	1918	1	K	1B
UK22	Looe, Cornwall^b^	England, UK	50.50383	− 4.60633	–	1901	1	E	1B
UK23	Nanstallon, Cornwall^a^	England, UK	50.47344	− 4.7692	–	2016	1	E	1B
UK24	Lanivet, Cornwall^a^	England, UK	50.45195	− 4.76513	–	2016	4	E	1B
UK25	Helligan and Grogley Woods, Cornwall^a^	England, UK	50.47995	− 4.79778	–	2016	2	E	1B
UK26	Polmorla, Cornwall^a^	England, UK	50.501	− 4.85685	–	2016	2	E	1B
UK27	Lampeter, Ceredigion^a^	Wales, UK	52.11126	− 4.07258	–	2016	2	E	1B
UK28	Rhosmaen, Carmarthenshire^a^	Wales, UK	51.92316	− 3.97646	–	2016	2	E	1B
UK29	Swansea Vale, Swansea^a^	Wales, UK	51.66526	− 3.9036	–	2016	2	E	1B
UK30	Clyne Valley, Swansea^a^	Wales, UK	51.59975	− 3.99761	–	2016	2	E, J	1B,2
UK31	Unknown^b^	UK	–	–	–	–	1	O	1A
IR01	Carragh, Kildare	Ireland	53.23044	− 6.72179	–	2016	2	E	1B
IR02	Bunclody, Wexford	Ireland	52.65607	− 6.65031	–	2016	2	A	1A
IR03	Kilkenny, Kilkenny	Ireland	52.65206	− 7.24265	–	2016	2	E, H	1B
**Native range**									
IN01	Tangmarg, Jammu and Kashmir^b^	India	34.0^d^	74.4^d^	1,829	1956	1	D	1B
IN02	Wangat Valley, Jammu and Kashmir^b^	India	34.3^d^	74.9^d^	–	1940	1	E	1B
IN03	Liddar Valley, Jammu and Kashmir^b^	India	33.7^d^	75.1^d^	3,048–3,353	1893	1	U	1A
IN04	Palgam, Jammu and Kashmir^b^	India	34.0^d^	75.3^d^	–	1913	1	V	1B
IN05	Sumadah Chun, Ladakh, Jammu and Kashmir^b^	India	33.8^d^	77.0^d^	4,115	1947	1	L	1A
IN06	Solang, Solang Valley, Himachal Pradesh	India	32.32293	77.15746	2,450	2009	1	R	1B
IN07	Rohtang, Kullu Valley, Himachal Pradesh^c^	India	32.32983	77.21162	3,067	2009	2	W	1B
IN08	Jibhi, Himachal Pradesh	India	31.57635	77.36196	1,938	2009	1	S	1B
IN09	Dimanjan Dogri, Baspa Valley, Himachal Pradesh^b^	India	31.3^d^	78.2^d^	3,810	1939	1	I	1B
IN10	Gauges Valley, Tihri-Gathwal^b^ Himachal Pradesh	India	30.7^d^	78.9^d^	1,829–2,134	1881	1	F	1B
IN11	Jammu and Kashmir^b^	India	–	–	–	1945	1	A	1A
PA01	Jalkhand, Khyber Pakhtunkhwa	Pakistan	34.97462	73.92942	3,098	2009	1	B	1A
PA02	Batakundi, Khyber Pakhtunkhwa	Pakistan	34.9337	73.75732	2,651	2009	1	A	1A
PA03	Lalazar, Khyber Pakhtunkhwa^c^	Pakistan	34.91687	73.76405	3,130	2009	1	B	1A
PA04	Naran Lake, Khyber Pakhtunkhwa	Pakistan	34.90726	73.67806	2,829	2009	1	T	1B
PA05	Naran, Khyber Pakhtunkhwa	Pakistan	34.86858	73.6088	2,300	2009	1	P	1A
PA06	Kaghan Valley^b^	Pakistan	34.5^d^	73.3^d^	–	1954	1	Q	1B
PA07	Thandiani, Hazara^b^	Pakistan	34.2^d^	73.3^d^	2,591	1956	1	P	1A

^a^Populations where the rust was released^14^.

^b^Samples were obtained from the Natural History Museum, UK.

^c^Populations where the rust was collected^14^.

^d^Data estimated based on location.

^e^Groups and subgroups were determined based on the phylogenetic analysis (Fig. 1).

**Figure 2:**
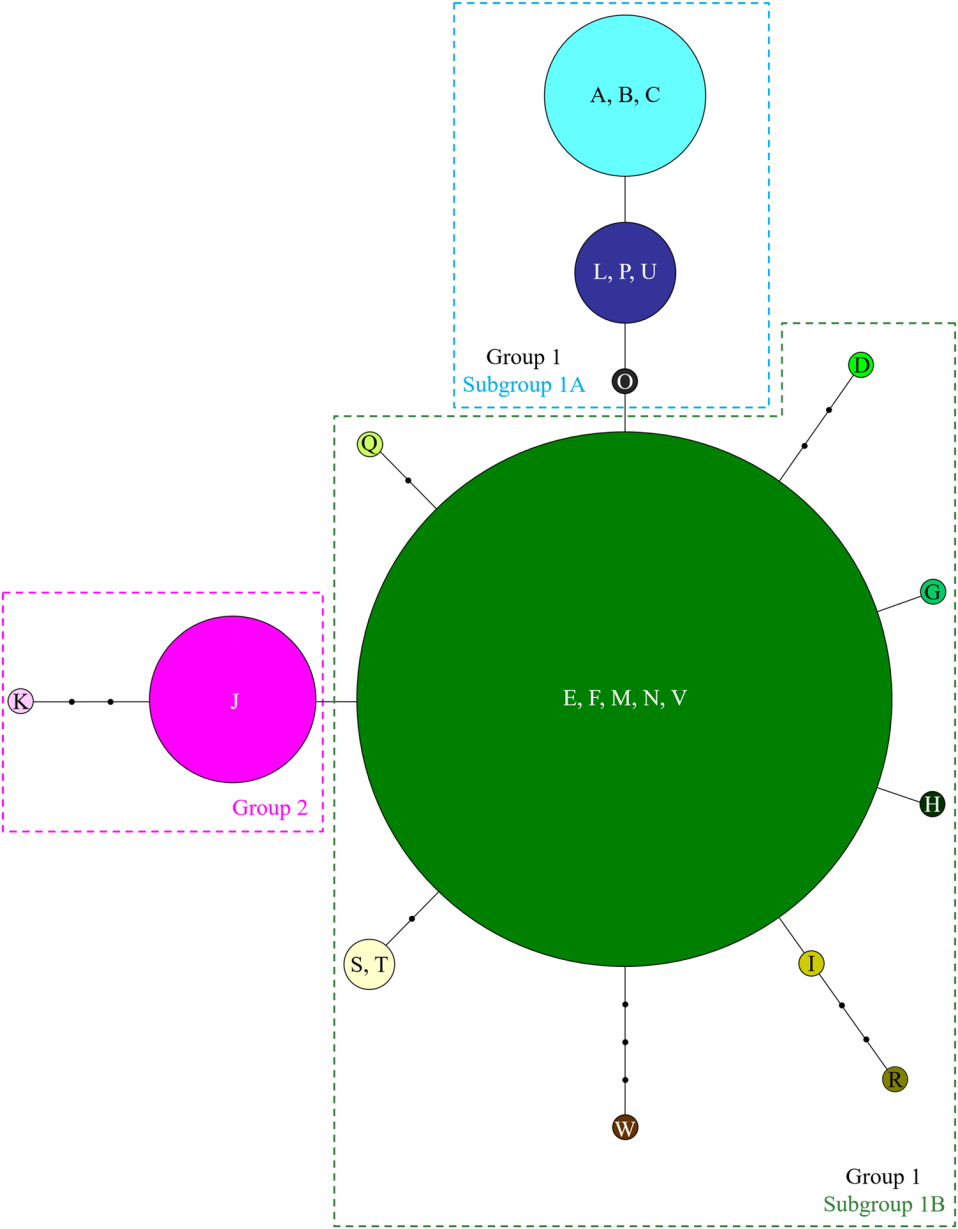
Parsimony network of chloroplast DNA haplotypes of *Impatiens glandulifera*. Each indel was treated as missing data in the analysis. Each link between haplotypes represents one mutational difference. Unlabelled nodes indicate inferred steps not found in the sampled populations. The size of each circle is roughly proportional to the haplotype frequency. The Groups indicated in the dashed boxes correspond to those in Fig. 1.

**Figure 3:**
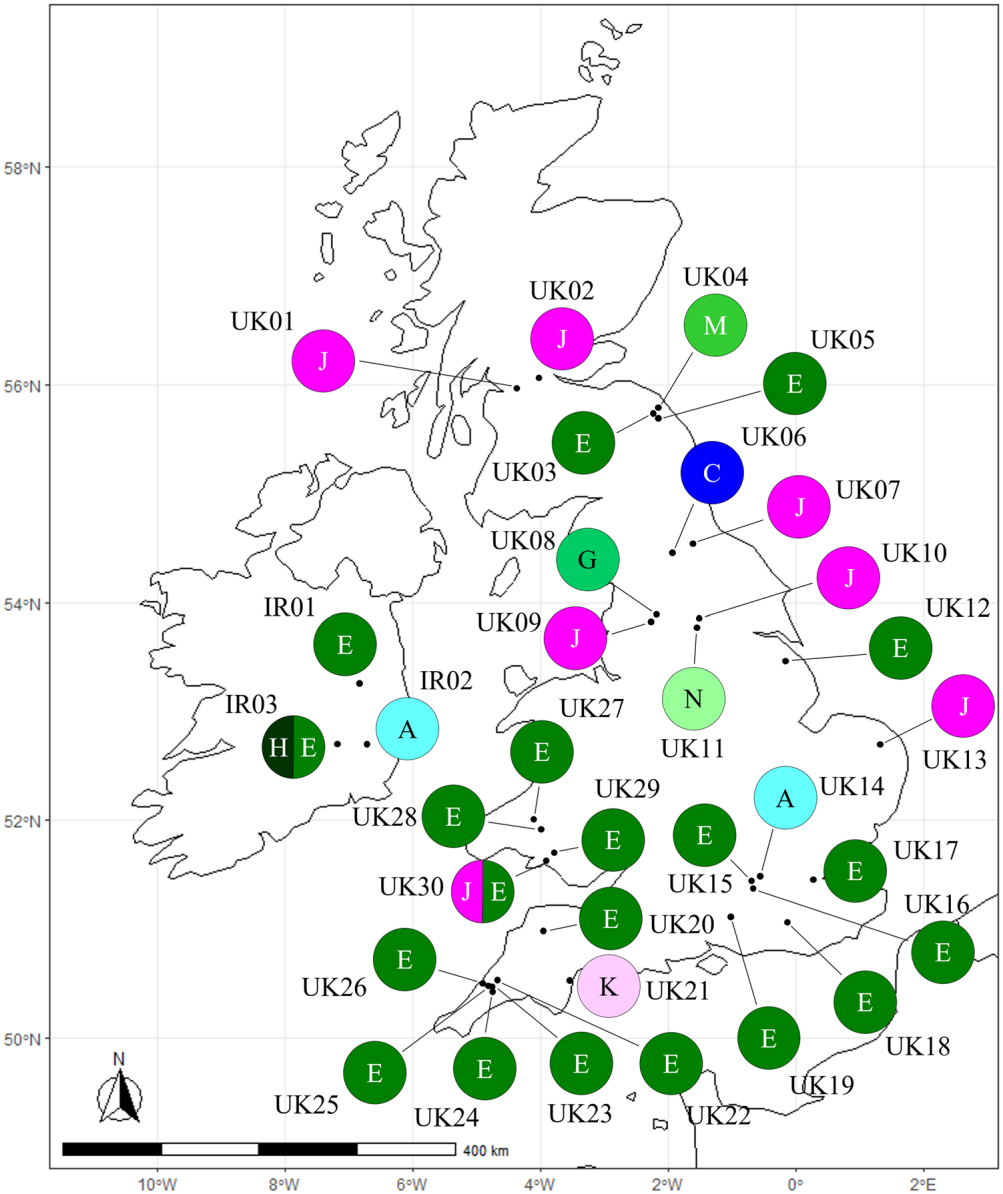
Geographical distribution of the chloroplast DNA haplotypes of *Impatiens glandulifera* in the introduced range. Numbers with the letter UK or IR correspond to the population numbers in Table 2. The letters in the circles correspond to the haplotypes in Figs. 1 and 2 and Tables 1 and 2. Haplotype O (UK31) is not included as its location is unknown. The map was generated using the ggplot2^34^, ggspatial^35^, sf^36^, rnaturalearth^37^ and rnaturalearthdata^38^ packages in R version 3.5.1^39^.

**Figure 4:**
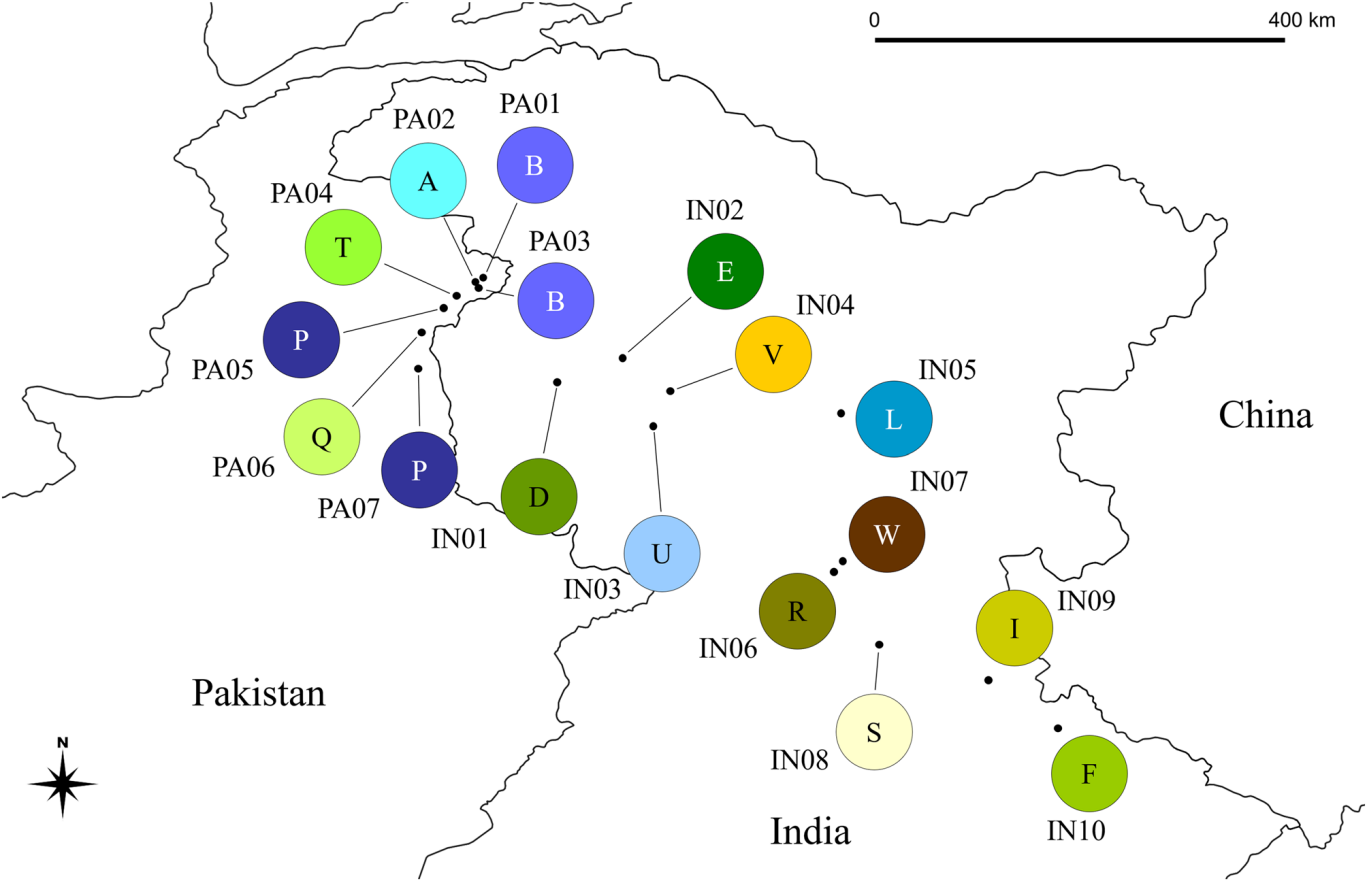
Geographical distribution of the chloroplast DNA haplotypes of *Impatiens glandulifera* in the native range. Numbers with the letter IN or PA correspond to the population numbers in Table 2. The letters in the circles correspond to the haplotypes in Figs. 1 and 2 and Tables 1 and 2. Haplotype A (IN11) is not included as its location is unknown. The map was created using QGIS 3.12.0^40^.

The unrooted network analysis of cpDNA using all haplotypes, in which indels were treated as missing data, is similar with the trees created from the ML and BI methods (Fig. 2). DNA sequences of ACCA in the positions 179–182 at *psbA-trnH* in haplotype W and those of GGATA in the positions 380–384 at *rpl32*-*trnL*^(UAG)^ in haplotype R were reverse complement of TGGT and TATCC, respectively, found in the other haplotypes (Table 1), and therefore these were treated as a single inversion event in the analysis. In this analysis, haplotypes A and L in Subgroup 1A are same as haplotypes B and C, and P and U, respectively. In Subgroup 1B, haplotype E is identical to haplotypes F, M, N, and V. Haplotype S is same as haplotype T. When each indel was treated as a fifth state in the analysis, homoplasy could be increased due to indels (see Supplementary Fig. S1). Based on both the network and phylogenetic analyses, Group 2, containing haplotypes J and K, is located in the outer branch and is likely to be the ancestral lineage of HB. In addition, the phylogenetic analyses suggest that the centre of diversification of HB in the native range is likely to be in the eastern part of the India Himalayas/Western Nepal, with the plant spreading and potentially evolving new genotypes in a north-westerly direction along the Himalayas through India and into Pakistan. Among the 23 cpDNA haplotypes, haplotype E was found to be the most widespread (Figs. 3, 4). Twenty of the 34 populations sampled in the BI region of the introduced range (18 of the 31 populations in the UK and two of the three populations in Ireland) and one of the 18 populations in the native range, Wangat Valley, India, were categorised as haplotype E. The geographical distributions for each haplotype, based on the cpDNA sequences, in the UK showed that haplotype E was most widespread in the south (Fig. 3). Haplotype J, the second most common haplotype, was found in seven populations, mainly concentrated in the north and east of the UK. Haplotype A was distributed in three populations in the introduced range (UK and Ireland) and in two populations in the native range including an unknown site in Kashmir, India. As would be expected in the native range, the haplotypes were found to be more diverse when compared with those in the introduced range (Fig. 4).

## Discussion

It is important to identify the native range of an invasive weed prior to the start of a CBC programme. However, the centre of origin of the invasive population within the native range can prove to be equally important, particularly if there are high levels of genetic diversity in the target plant in its invasive range. Molecular methods are one of the tools that can be employed to determine the origin of introduced weeds, particularly when they have a wide native range. The success rate of CBC programmes has been shown to significantly improve when natural enemies are collected from populations in the native range which genetically match those in the introduced range^41^.

CpDNA is inherited maternally^42^ and lacks recombination^43^. Therefore, data from cpDNA can provide important insights into evolutionary processes in plants such as hybridisation, population structure and phylogeography^44^. A total of 23 haplotypes of HB were found during this study across the introduced and native ranges. Ten of these HB haplotypes were distributed across the 34 populations in the BI and 15 in the 18 native populations. These results corroborate those of Hagenblad et al.^29^ and Nagy and Korpelainen^30^, demonstrating that there is more variation in HB in the native range than in the introduced range. HB is native to the foothills of the Himalayas, with populations of the weed occurring in valleys separated by mountain ranges. As a result, populations have evolved in isolation and as shown here, represent different haplotypes.

This study demonstrates that haplotypes of HB in the BI and the native range form two distinct genetic groups: Group 1, which can be divided into two subgroups; Subgroup 1A contains two haplotypes which are present in England and Ireland (A and C); Subgroup 1B contains seven haplotypes which are present in the BI (E, G, H, J, K, M, N). Haplotype E is the most common genotype found in the BI and is particularly prevalent in southern England and Wales. Haplotypes from the native range could be divided between the two subgroups, with the majority from Pakistan belonging to Subgroup 1A and those from India to Subgroup 1B. Group 2 consists of two haplotypes which are present in the UK (J and K), but as yet have not been matched to haplotypes in the native range. Haplotype J is predominately distributed in the northern and eastern parts of England and in Scotland. The earliest herbarium samples provided by the Natural History Museum, dating back to before the First World War, gave particular insight into the introduction of HB into the BI. Stately homes and country residences of the aristocracy were significant growers of new plant species brought to the BI by plant hunters and botanists^45^ such as John Forbes Royle who described *I. glandulifera*. It is likely that HB would have first been grown on these estates, and indeed the plant can be found in many of them today. These historical samples dating back more than 100 years are likely to represent genotypes with very little genetic divergence from the initial founder populations. Interestingly, our analyses placed these oldest samples into haplotypes from both genetic groups, and the two subgroups suggesting that HB was introduced from the native range to the BI on at least two, but probably three separate occasions. These herbarium specimens, in addition to those from the native range, can provide significant evolutionary insights into the species, as they represent a genetic snapshot at a particular time and place^46^.

In Group 1, it was possible to match both subgroups to populations in the native range. Subgroup 1A had an identical cpDNA profile (haplotype A) to two populations in the Batakundi, Kaghan Valley of Pakistan (PA02) and in an unknown location in Jammu and Kashmir of India (IN11); whilst Subgroup 1B had an identical cpDNA profile (haplotype E) to the population analysed from the Wangat Valley in Kashmir (IN02). As for those sites with full information, these two regions between the Kaghan Valley and the Wangat Valley are geographically distinct from each other, separated by the Pir Panjal range and Nanga Parbat region, which are high mountain ranges that divide the Vale of Kashmir in India and the North-West Province of Pakistan. However, Group 2 which consists of the haplotypes J and K was not associated with any of the populations included in the study from the native range. The position of Group 2 containing the haplotypes J and K, in the phylogenetic tree (closest to the outgroup) indicates that these haplotypes may be an ancestral lineage and it is possible that Groups 1 and 2 have diversified from north-eastern India and potentially north-western Nepal (the most easterly part of the native range of Himalayan balsam). The theory of the diversification of *I. glandulifera* along the mountain slopes of Nepal and India towards Pakistan is supported by a study by Janssens et al.^47^; who concluded that the centre of origin for *Impatiens* species is south-west China. In order to confirm this, additional samples collected more widely from the native range, especially from Nepal, should be included in future studies. In addition, to confirm whether the haplotypes J and K represent an ancestral lineage, due to the low bootstrap value in the phylogenetic analysis shown in this study, other methods such as inter-simple sequence repeats PCR or next-generation sequencing should be used.

The results of this study provide an insight into the genetic diversity of HB in the BI and yield crucial data concerning where to focus future searches for additional compatible strains of the rust *P. komarovii* var. *glanduliferae* in the native range of the species. In a mountainous region such as the Himalayas, it is probable that the rust has evolved with distinct plant biotypes in isolation and that, as such, distinct strains or pathotypes of the rust exist^48, 49^. Consequently, the potential for intraspecies specificity of biological control agents, particularly co-evolved, biotrophic pathogens such as *P. komarovii* var. *glanduliferae*, is significant. The results of the releases of two strains of this rust in England and Wales from the Kullu Valley of India (IN07) and the Kaghan Valley of Pakistan (PA03), have demonstrated the need to consider the introduction of more strains of the rust, since there are weed populations resistant to both strains in the BI^14^. There are numerous examples where matching the genotypes of a target invasive alien weed with the isolate of a fungal biological control agent has proven to be critical: most notably, control of rush skeleton weed, *Chondrilla juncea* in Australia^41^. In this example, a strain of the rust *Puccinia chondrillina* collected from Italy, was released and had a severe impact on one of three forms of the weed in the field; with populations levels being decreased by up to 99%^50, 51^. Unfortunately, as not all forms were targeted, due to them not being recognised initially as distinct morphotypes, the distribution of the two other forms increased significantly. This necessitated the need to introduce additional rust strains, more virulent towards the resistant forms of the plants^52^ to achieve successful control^41^. This case illustrates clearly the possibility to counter the presence of natural resistance in weed populations by the introduction of new pathogen strains^53^. There are, however, other instances where this appears not to be a factor for successful control, for example, mistflower, *Ageratina riparia*, in Australia, New Zealand and Hawaii^54^. In some cases, an isolate of a pathogen has broad intraspecies specificity, independent of where it was collected from^55^. The susceptibility of HB genotypes to strains of the rust, however, is not clear cut; the rust strain originally collected from India was from the centre location of the native range (IN07), an area where the molecular evidence reported here indicates that none of the BI haplotypes originated. However, this rust strain is able to infect some of the BI populations in England and Wales and has established in the field at a site where haplotype E is the dominant type (UK18)^14^. The release of the rust strain from Pakistan has resulted in the infection of a separate cohort of HB populations^14^, enhancing the success of the CBC programme. In the case of HB, it will be advisable to collect a range of rust strains from throughout the native range, in addition to focusing on the areas matching the likely origin of a plant genotype. Potentially, this will also create more genetic diversity within the introduced populations of the rust making local adaptations to evolving HB populations more likely and rapid^56^.

The sample number used in this study was limited and the number of plants sampled at each site in the BI was low, and may have masked a more significant number of mixed haplotypes at the sites. Only at two sites were the two haplotypes found (UK30 and IR03) and at the four sites where 4–8 plants were sampled only one haplotype was revealed. The results of this research have confirmed previous findings that populations of *I. glandulifera* in the BI have been introduced from both India and Pakistan^30^. The samples from the BI can be organised into two groups, one being composed of two subgroups, based on the sequences of the six cpDNA regions. Although caution should be taken when interpreting these results, they do provide compelling evidence where searches to find additional strains of the rust that are fully compatible with the dominant haplotypes in the BI should be targeted. Of the two native range haplotypes present in the introduced range, one is from the Butakundi, Kaghan Valley (Pakistan) and positioned within Subgroup 1A, whilst the other, from the Wangat Valley, Kashmir (India) is positioned in Subgroup 1B. No haplotypes were found amongst the native range samples that matched UK haplotypes J and K from Group 2. However, the position of the haplotypes J and K on the phylogenetic tree suggests that these haplotypes may be found in the most easterly part of the native range (where India borders Nepal). Thus, this region may yield rust strains compatible with the HB haplotypes J and K, and should, therefore, also be targeted in future surveys. These additional rust strains would thereby enhance the likelihood of successful biological control of HB in the BI.

## Methods

### Plant material

#### British Isles (BI)

A total of 34 distinct HB populations, including some rust-release sites, were used in this study from across the BI (Table 2). The majority of the leaf samples (26 populations) were collected in 2016 from living plants, dried immediately in silica gel and stored at 4 °C, using the techniques described by Gaskin et al.^18^. The number of plants sampled from each population (*N*) varied from one to eight; although only for three populations were single samples available (UK03, River Till, Northumberland; UK05, River Tweed, Northumberland; and UK23, Nanstallon, Cornwall). For 23 populations, leaf samples were collected from a minimum of two individual plants. In order to check the validity of using limited sample numbers at each site, eight plants were sampled from four separate parts of the HB infestation at Harmondsworth Moor, Middlesex (UK14); and four plants from Silwood Park, Berkshire (UK15) and also Lanivet, Cornwall (UK24).

Leaf samples from seven sites in England were provided by the Natural History Museum (London, UK) from historic herbarium material dating back to before the end of the First World War; collection dates are from 1898 to 1945 (and one sample from the UK from an unknown date and location). The aim was to maximise the chance of obtaining samples from the original introductions, before HB became widespread.

#### Himalayan native range

Eighteen HB populations from the native range (11 from India and seven from Pakistan) were also included in the study (Table 2). Leaf samples from eight populations were taken from CABI’s fungal herbarium material that had been collected during surveys to look for natural enemies of HB from 2006–2010. Consequently, leaf material was only available from one plant, apart from at Rohtang, Himachal Pradesh, India (IN07) where two plants were available. The herbarium samples had been dried in a plant press and stored in wax packets at room temperature. The remaining 10 herbarium samples were obtained from the Natural History Museum. These samples included eight Indian samples and two from Pakistan collected 50–100 years ago. In addition, in order to use *I. parviflora* as an outgroup species for phylogenetic analyses, the leaf samples of this species were collected from one plant in Egham (Surrey, UK).

### DNA extraction, PCR amplification and sequencing

Total genomic DNA was extracted from 10–20 mg of dried leaf tissue, depending on the availability, particularly for the herbarium samples, using DNeasy Plant Mini Kit (Qiagen, Hilden, Germany) or Power Plant Pro DNA Isolation Kit (MO BIO Laboratories Inc., West Carlsbad, USA). Preliminary screening of the rDNA-ITS region as well as 15 non-coding regions of cpDNA using three individuals from geographically distant HB populations (UK, India and Pakistan) observed polymorphisms in six regions of cpDNA, *trnL-trnF* (*trnL* intron, and *trnL* [UAA] 3′ exon-*trnF* [GAA] intergenic spacer), *atpB*-*rbcL* intergenic spacer, *rps16* Intron, *trnG* Intron, *psbA*-*trnH*^(GUG)^ intergenic spacer and *rpl32*-*trnL*^(UAG)^ intergenic spacer (see Supplementary Table S1). Consequently, these six cpDNA fragments were amplified and sequenced for all samples from the introduced and native range. PCR amplifications were undertaken in reaction volumes of 20 μl containing 10 ng of genomic DNA templates, 10 μl of MegaMix-Royal (Microzone, Haywards, UK) and 0.5 μM of each primer or the same reaction volumes containing of 10 ng of genomic DNA templates, 0.5 U KOD Hot Start DNA polymerase (Novagen/Toyobo, Darmstadt, Germany), 1 × PCR Buffer, 0.2 mM of dNTPs, 1.0 mM MgSO_4_ and 0.3 μM of each primer. DNA amplification was performed in a Mastercycler (Eppendorpf AG, Hamburg, Germany). The PCR conditions were as follows: denaturation at 96 °C for 5 min, followed by 36 cycles of 94 °C for 45 s, 50–60 °C (depending on the primers) for 30 s, and 72 °C for 90 s. PCR products were fractionated in 1.5% (w/v) agarose gels, and visualised using SafeView Nucleic Acid Stain (NBS Biologicals, Huntingdon, UK) and UV illumination. The PCR products were purified with MicroCLEAN (Microzone, Haywards, UK) and directly sequenced bidirectionally using an ABI 3100 Genetic Analyzer (Applied Biosystems, Tokyo, Japan) with a Big Dye Terminator Cycle Sequencing Ready Reaction Kit (Applied Biosystems, Austin, USA) with the same primers used for PCR. All haplotype sequences were deposited in DDBJ/EMBL/GenBank under the accession number LC379653-LC379796.

### Phylogenetic analysis

Sequence alignments were generated using the MUSCLE algorithm in the program MEGA 7.0.14^57^ and manually optimised. In order to confirm that *I. parviflora* became the outgroup species, the sequence of *C. controversa* (Cornaceae), designated as one of the outgroup species, was obtained from the GenBank database. ML and BIf analyses were performed using RAxML version 8.2.9^58^ and MrBayes version 3.2.6^59^, respectively, for both the individual data partitions as well as the combined aligned dataset. The best fit nucleotide substitution model was determined using Kakusan 4^60^, which also generates input files for ML and BIf. Best fit models were evaluated using the Akaike Information Criterion (AIC)^61^ for ML and the Bayesian Information Criterion (BIC) with significant determined by Chi-square analysis. Equalrate model among regions with GTR + G in AIC and non-partitioned model among regions in BIC were selected. In the ML analyses, the resultant tree was evaluated by bootstrap analysis with 1,000 replicates. In the BIf analyses, two independent runs, each with four Markov chain Monte Carlo were run for 3,000,000 generations. Samples were taken every 100 generations. Burn-in values were estimated using Tracer v1.6^62^. The first 25% of generations were discarded as burn-in and then a 50% majority rule consensus tree was generated from the sampled trees. Phylogenetic trees outputted from the ML and BIf analyses were processed via FigTree v1.4.3^63^. A network tree of the combined cpDNA regions was constructed with a statistical parsimony network using TCS v. 1.21^64^. Insertions of 5 bp observed at *rps16* Intron and *psbA-trnH* were considered as single mutation events. The analyses were conducted twice, in which each indel was treated as missing data (Fig. 2) and a fifth state (see Supplementary Fig. S1).

## Supplementary information

Supplementary file1 (docx 346 kb)

## Data Availability

Sequence data were deposited in DDBJ/EMBL/GenBank under the accession number LC379653-LC379796.
