# Supplementary material for: Chloroplast DNA analysis of the invasive weed, Himalayan balsam (Impatiens glandulifera), in the British Isles
Source: Sci Rep. 2020 Jul 3;10:10966. doi: 10.1038/s41598-020-67871-0 (PMC7335094; doi:10.1038/s41598-020-67871-0)
Supplement: Supplementary file 1 — Supplementary file1 (docx 346 kb) [file 41598_2020_67871_MOESM1_ESM.docx]

Chloroplast DNA analysis of the invasive Himalayan balsam (*Impatiens glandulifera*) in the British Isles

Daisuke Kurose*, Kathryn M. Pollard, Carol A. Ellison†

CABI-UK

Bakeham Lane, Egham, Surrey TW20 9TY, UK

Corresponding author

D. Kurose

Email: D.Kurose@cabi.org

†Deceased 19 April 2020

| **Table S1** Primers used in this study, with sequences and references | | | |
| --- | --- | --- | --- |
| Locus | Primer name | Primer sequence (5'-3') | Reference |
| *trnL-trnF* | trnLc | CGAAATCGGTAGACGCTACG | Taberlet et al. (1991)^1^ |
|  | trnLd | GGGGATAGAGGGACTTGAAC | Taberlet et al. (1991)^1^ |
|  | trnLe | GGTTCAAGTCCCTCTATCCC | Taberlet et al. (1991)^1^ |
|  | trnFf | ATTTGAACTGGTGACACGAG | Taberlet et al. (1991)^1^ |
| *atpB-rbcL* | IMP-atpB | ACATCTAGTACCGGACCAATGA | Janssens et al. (2006)^2^ |
|  | IMP-rbcL | AACACCAGCTTTGAATCCAA | Janssens et al. (2006)^2^ |
| *rps16* intron | rps16 IntronF | ATCGAACATCAATTGCAACG | Scarcelli et al. (2011)^3^ |
|  | rps16 IntronR | TAGAAAGCAACGTGCGACTT | Scarcelli et al. (2011)^3^ |
| *trnG* intron | trnG IntronF | GCGGGTATAGTTTAGTGGTAA | Scarcelli et al. (2011)^3^ |
|  | trnG IntronR | GCTTGGAAGGCTAGGGGTTA | Scarcelli et al. (2011)^3^ |
| *psbA-trnH*^(GUG)^ | psbAF | GTTATGCATGAACGTAATGCTC | Sang et al. (1997)^4^ |
|  | trnHF | CGCGCATGGTGGATTCACAATCC | Sang et al. (1997)^4^ |
| *rpl32-trnL*^(UAG)^ | rpL32-F | CAGTTCCAAAAAAACGTACTTC | Shaw et al. (2007)^5^ |
|  | trnL^(UAG)^ | CTGCTTCCTAAGAGCAGCGT | Shaw et al. (2007)^5^ |


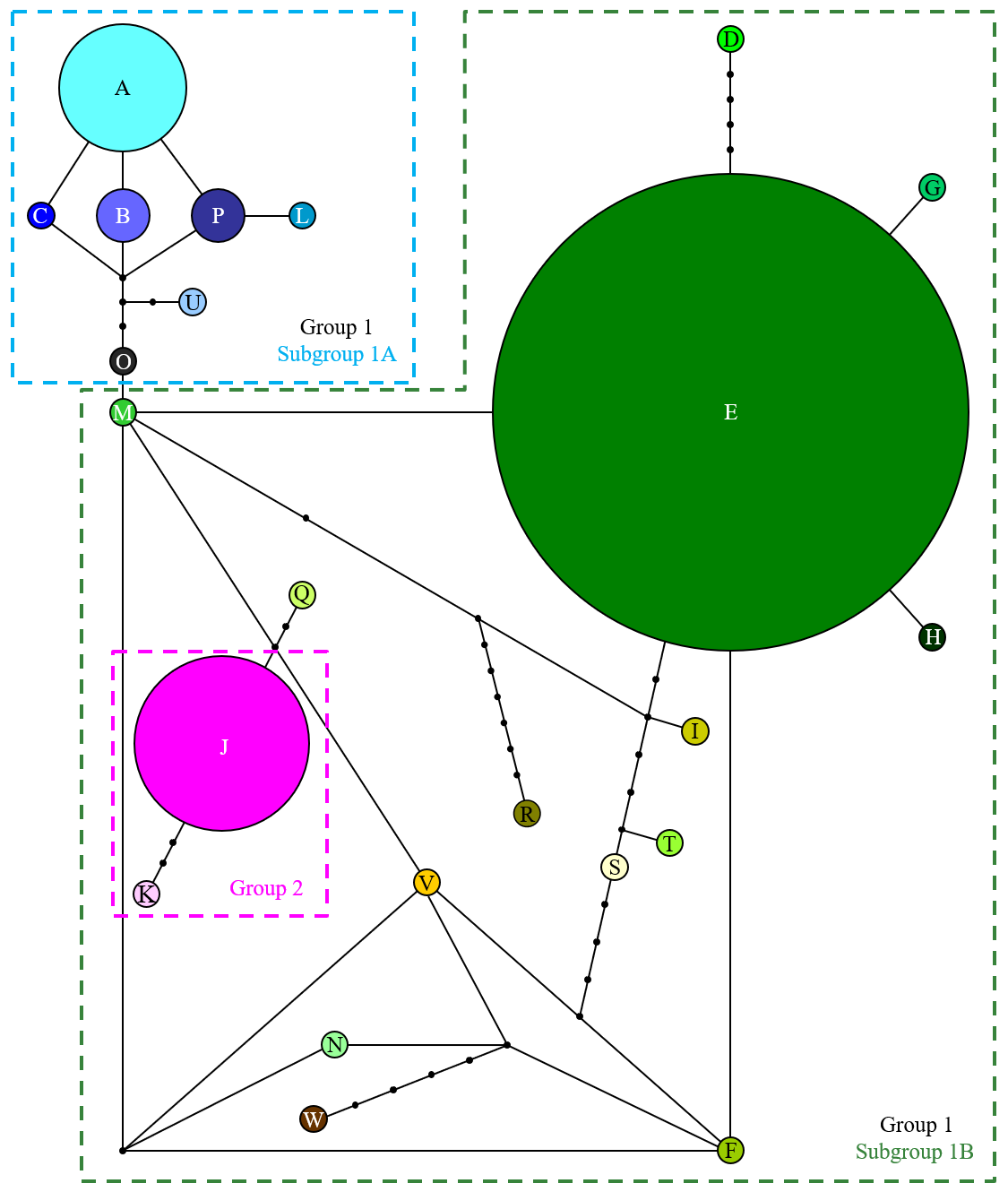
 **Figure S1.** Parsimony network of chloroplast DNA haplotypes of *Impatiens glandulifera*. Each indel was treated as a fifth state in the analysis. Each link between haplotypes represents one mutational difference. Unlabelled nodes indicate inferred steps not found in the sampled populations. The size of each circle is roughly proportional to the haplotype frequency. The Groups indicated in the dashed boxes correspond to those in Fig. 1.

**Reference**

1 Taberlet, P., Gielly, L., Pautou, G. & Bouvet, J. Universal primers for amplification of three non-coding regions of chloroplast DNA. *Plant Mol. Biol.* **17**, 1105-1109, doi.org/10.1007/BF00037152 (1991).

2 Janssens, S. *et al.* Phylogenetics of *Impatiens* and *Hydrocera* (Balsaminaceae) using chloroplast *atpB-rbcL* spacer sequences. *Syst. Bot.* **31**, 171-180, doi.org/10.1600/036364406775971796 (2006).

3 Scarcelli, N. *et al.* A set of 100 chloroplast DNA primer pairs to study population genetics and phylogeny in monocotyledons. *PLoS One* **6**, e19954, 10.1371/journal.pone.0019954 (2011).

4 Sang, T., Crawford, D. J. & Stuessy, T. F. Chloroplast DNA phylogeny, reticulate evolution, and biogeography of *Paeonia* (Paeoniaceae). *Am. J. Bot.* **84**, 1120-1136, doi.org/10.2307/2446155 (1997).

5 Shaw, J., Lickey, E. B., Schilling, E. E. & Small, R. L. Comparison of whole chloroplast genome sequences to choose noncoding regions for phylogenetic studies in angiosperms: the tortoise and the hare III. *Am. J. Bot.* **94**, 275-288, doi.org/10.3732/ajb.94.3.275 (2007).
